# Serum proteomics and metabolomics reveal novel noninvasive molecular signatures for hepatocellular carcinoma diagnosis

**DOI:** 10.3389/fonc.2026.1797408

**Published:** 2026-04-22

**Authors:** Lin Lin, Weiquan You, Zhaopei Guo, Lu Lai, Xiangjun Tang, Yan Pan, Yutong Li, Qishui Ou, Ya Fu

**Affiliations:** 1 Department of Laboratory Medicine, The First Affiliated Hospital, Fujian Medical University, Fuzhou, China; 2 Clinical Laboratory Diagnostics, The First Clinical College, Fujian Medical University, Fuzhou, China; 3 The School of Public Health, Fujian Medical University, Fuzhou, China; 4 Fujian Key Laboratory of Laboratory Medicine, The First Affiliated Hospital, Fujian Medical University, Fuzhou, China; 5 Gene Diagnosis Research Center, Fujian Medical University, Fuzhou, China; 6 Fujian Clinical Research Center for Clinical Immunology Laboratory Test, The First Affiliated Hospital, Fujian Medical University, Fuzhou, China; 7 Department of Laboratory Medicine, National Reginal Medical Center, Binhai Campus of the First Affiliated Hospital, Fujian Medical University, Fuzhou, China

**Keywords:** biomarker, diagnosis, hepatocellular carcinoma, metabolites, proteomics, serum

## Abstract

**Objective:**

This study aimed to identify and validate noninvasive serum protein and metabolite biomarkers for the early detection and prognostic assessment of hepatocellular carcinoma (HCC).

**Methods:**

In this study, we systematically analyzed serum proteomics and metabolomics across four cohorts, including healthy controls (Health) and patients with chronic hepatitis B (CHB), liver cirrhosis (LC), and HCC. Standardized sample handling protocols were applied, and high-resolution mass spectrometry combined with rigorous statistical analyses was used to identify differential proteins and metabolites.

**Results:**

Our analyses indicated that GRHPR was able to distinguish HCC from non-HCC conditions. GRHPR demonstrated good individual diagnostic performance, with an area under the receiver operating characteristic curve (AUC) of 0.936. Moreover, a combined biomarker panel incorporating GRHPR and isobutyric acid achieved an AUC of 0.988. After internal validation using bootstrap resampling, the corrected AUC was 0.978, suggesting that the model retained favorable discriminatory performance. Collectively, these findings support the potential feasibility of a non-invasive, serum-based diagnostic signature that integrates proteomic and metabolomic data for the early detection of HCC.

**Conclusion:**

In summary, this study suggests that specific serum proteins and metabolites may facilitate the detection of hepatocellular carcinoma. GRHPR and isobutyric acid showed relatively strong diagnostic performance, and GRHPR may also have prognostic value. The combined panel demonstrated improved discriminatory ability. Together, these findings support the potential utility of integrating proteomic and metabolomic markers as a non-invasive approach for HCC identification.

## 1 Introduction

HCC is the predominant form of primary liver cancer, accounting for 70% to 85% of all cases (1, 2). It most commonly develops in chronic liver disease and cirrhosis. The main etiological factors include chronic hepatitis virus infection, alcohol-related liver disease, and metabolic dysfunction associated steatotic liver disease (3–6). Its molecular heterogeneity and often silent clinical course contribute to late presentation and poor outcomes (7, 8). In this context, improving early detection remains a central objective of HCC research when potentially curative treatments are feasible.

Despite the widespread use of imaging-based surveillance and serological tests, there are notable shortcomings in current diagnostic strategies. The performance of ultrasound depends on the operator and is reduced in nodular cirrhotic livers (9). Conventional serum markers such as alpha-fetoprotein (AFP) lack sufficient sensitivity and specificity, particularly for early-stage tumors and across heterogeneous patient populations (10, 11). These limitations highlight the urgent need to discover and validate new, reliable and non-invasive biomarkers.

To address this need, we profiled the serum proteomics and metabolomics of Health, CHB, LC, and HCC populations, aiming to delineate molecular features that can distinguish HCC from non-HCC. Our objective was to identify and prioritize candidate serum biomarkers and composite panels with diagnostic potential from these data.

## 2 Methods

### 2.1 Patients and serum samples

To investigate potential biomarkers for the early diagnosis of HCC, serum samples were collected from 4 healthy controls, 8 patients with CHB, 7 patients with LC, and 9 patients with HCC at the First Affiliated Hospital of Fujian Medical University between 2021 and 2022. The inclusion criteria for the HCC group were as follows: (1) histopathological confirmation of HCC; (2) undergoing curative hepatectomy; (3) no prior antitumor treatment before surgery; (4) availability of comprehensive clinicopathological data. For the Health, CHB, and LC groups, diagnoses were based on established physiological parameters and imaging findings. The sex distribution and detailed physiological and biochemical indices of all participants, as well as the clinicopathological features of the HCC cohort, are presented in **Supplementary Table S1** and **Supplementary Table S2**.

### 2.2 Clinical laboratory measurements

ALT and AST were quantified using Cobas8000 (Roche, Basel, Switzerland). Hepatitis B surface antigen, hepatitis B e antigen and AFP were detected using the Alinity system (Abbott, Illinois, USA). Protein Induced by Vitamin K Absence or Antagonist-II (PIVKA-II) was detected using G12000 (Fujirebio, Tokyo, Japan).

### 2.3 Serum proteomics analysis

After protein extraction and enzymatic digestion, serum samples were analyzed on a TimsTOF mass (Bruker, Massachusetts, Daltonics), connected to an Evosep One system liquid chromatography (Evosep, Odense, Denmark) in the data-independent acquisition mode. Bioinformatics analyses were performed on the resulting data following acquisition.

### 2.4 Serum metabolomics profiling

After sample extraction, metabolites were separated using a 1290 Infinity LC ultra-high-performance liquid chromatography (UHPLC) system (Agilent Technologies, California, USA) equipped with a column (waters, Ireland), and subsequently analyzed with a TripleTOF 6600 mass spectrometer (AB Sciex, Ontario, Canada). Data acquisition was performed in both positive and negative electrospray ionization (ESI) modes. The acquired datasets were then subjected to metabolite identification, quality assessment of the experimental data, and finally comprehensive statistical analysis.

### 2.5 Statistical analysis

Data analysis and statistical visualization were conducted using GraphPad Prism software (version 9.0). Protein expression differences between the hepatocellular carcinoma (HCC) group and the non-HCC group were analyzed using the Student’s t-test. Protein levels were log-transformed when appropriate. Fold changes (FC) were calculated, and proteins with |log_1.5_(FC)| > 1 and P < 0.05 were considered significantly differentially expressed. The expression of GRHPR was further validated using Clinical Proteomic Tumor Analysis Consortium (CPTAC) proteomics data from the Human Protein Atlas (HPA).

Metabolite differences between the HCC and non-HCC groups were analyzed using the Student’s t-test after log-transformation when appropriate, and FC values were calculated; VIP scores from the OPLS-DA model were used to evaluate metabolite contributions, with differential metabolites defined as those meeting the criteria of VIP > 1, adjusted P < 0.05, and |log_1.5_(FC)| > 1.

Samples were divided into four groups (Health, CHB, LC and HCC) for further analyses. Fuzzy c-means clustering was performed to identify dynamic expression trends, and relevant clusters were intersected with the differentially expressed proteins to obtain candidate biomarkers. For two-group comparisons, normality was assessed prior to testing. The Student’s t-test or Mann–Whitney U test was applied as appropriate. All tests were two-sided, and this approach was used consistently throughout the study unless otherwise specified.

Functional enrichment analyses, including Gene Ontology (GO) annotation and Kyoto Encyclopedia of Genes and Genomes (KEGG) pathway analysis, were conducted using R package software (version 3.6). Gene set enrichment analysis (GSEA) was performed with the GSEA software (https://www.gsea-msigdb.org/gsea/login.jsp). Protein-protein interaction (PPI) data for the target proteins were obtained from STRING (https://string-db.org/), and interaction networks were visualized and further analyzed using Cytoscape software (version 3.7.2). The node degree of each protein was calculated to assess its functional significance within the PPI network. Bootstrap ROC curves and survival analyses were performed using R software.

## 3 Results

### 3.1 Serum proteomic characterization of healthy, CHB, LC, and HCC populations

**Figure 1:**
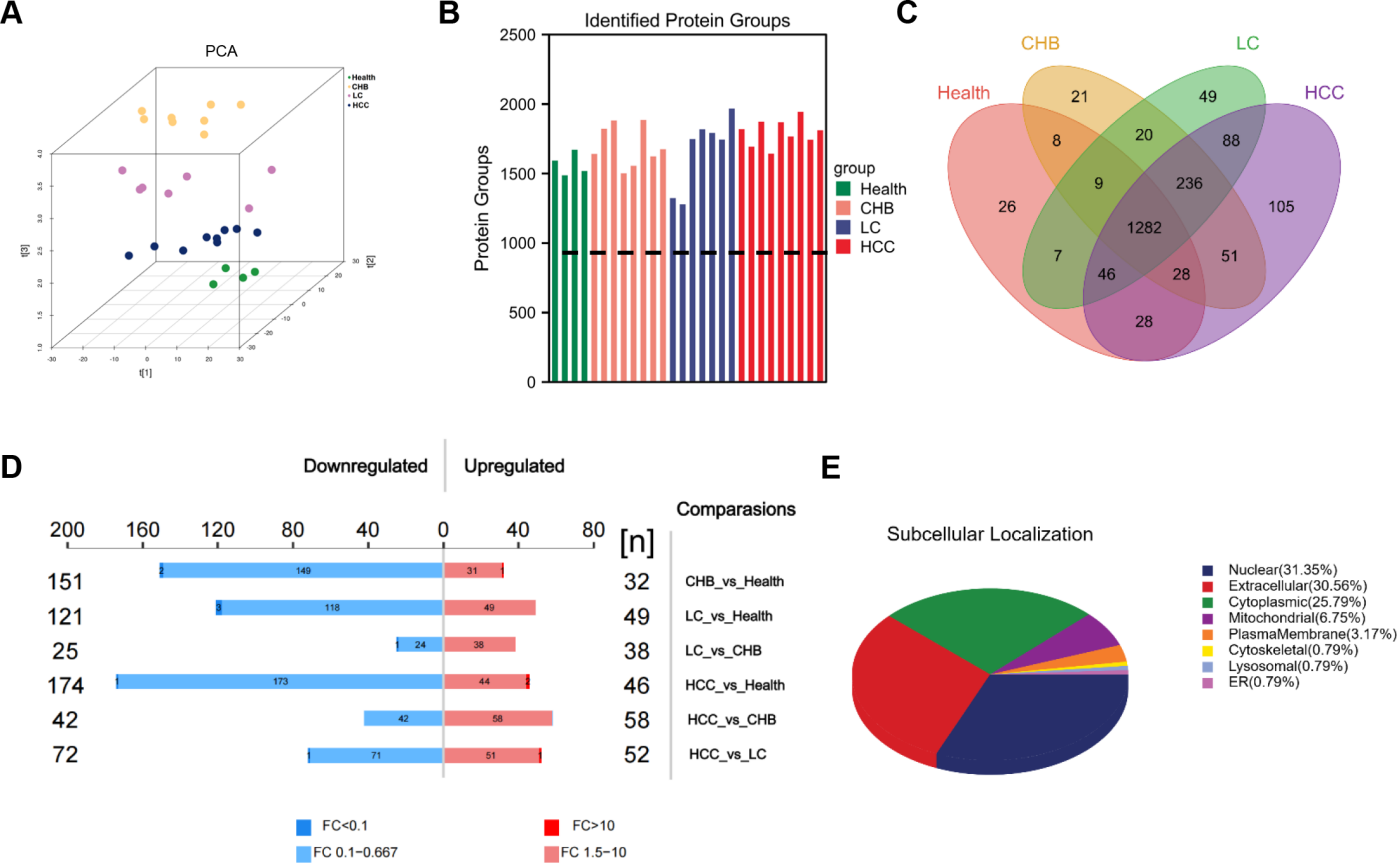
Research overview and characterization of serum proteome. **(A)** 3D PCA distribution map of all samples. **(B)** DIA identification result statistics bar chart. **(C)** Venn diagram of samples between groups. **(D)** Bar chart of quantitative differences in proteins. **(E)** Pie chart of subcellular localization distribution of differentially expressed proteins.

In this study, a total of 28 serum samples were collected, including 4 control samples, 8 samples from patients with CHB, 7 from patients with LC, and 9 from patients with HCC. Three-dimensional principal component analysis (3D-PCA) was performed on all samples, revealing a clear separation of the four groups in the PCA plot, which suggests distinct protein expression profiles across the groups (**Figure 1A**). A total of 2240 proteins were identified, with the number of proteins detected in each group summarized in **Figure 1B**. To further assess the overlap in protein identification among the groups, shared and unique proteins were visualized using a Venn diagram (**Figure 1C**). Differentially expressed proteins across the groups (DEPs, |log_1.5_(FC)| >1, *P* < 0.05) are displayed in **Figure 1D**. Subcellular localization analysis was also conducted for all DEPs, and the distribution of these proteins across cellular organelles is illustrated in **Figure 1E**.

### 3.2 Differential protein analysis and pathway enrichment

**Figure 2:**
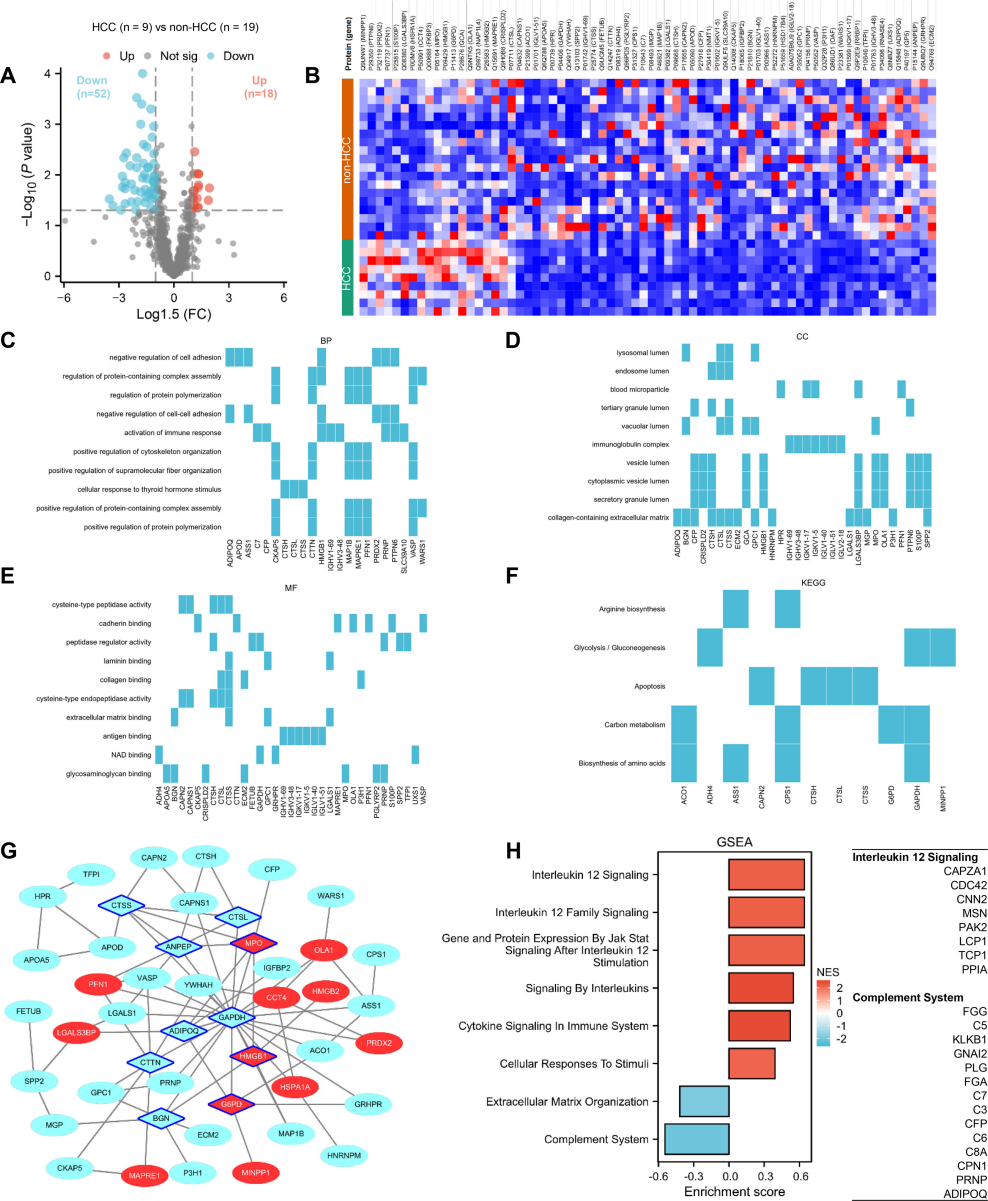
Difference in serum proteome between HCC and non-HCC groups. **(A)** Volcano plot of serum proteomic profiles in HCC vs non-HCC. **(B)** Heatmap of serum proteomic profiles in the two groups. **(C)** BP pathway analyses with DEPs. **(D)** CC pathway analyses with DEPs. **(E)** MF pathway analyses with DEPs. **(F)** KEGG pathway analyses with DEPs. **(G)** PPI network analysis of the interactions between the two groups. **(H)** GSEA pathway analyses in two groups.

The 28 samples were categorized into HCC and non-HCC groups. Volcano plot analysis revealed 18 proteins that were upregulated and 52 proteins that were downregulated in the HCC group compared to the non-HCC group (|log_1.5_(FC)| >1, *P* < 0.05) (**Figure 2A**). The result of differential expressed proteins (DEPs) was visualized as a heatmap which clearly distinguished between the two groups (**Figure 2B**).

The GO Biological Process (BP) analysis indicated that the DEPs were primarily enriched in the negative regulation of cell adhesion, regulation of protein-containing complex assembly and polymerization, activation of immune response, and positive regulation of cytoskeleton and supramolecular fiber organization. In addition, cellular responses to thyroid hormone stimulus were also observed, suggesting potential involvement in hormonal signaling modulation (**Figure 2C**). The GO Cellular Component (CC) analysis demonstrated that these proteins were predominantly localized to luminal compartments, including the lysosomal lumen, endosome lumen, vesicle lumen, secretory granule lumen, and vacuolar lumen, as well as blood microparticles and immunoglobulin complexes. Enrichment in the protein-containing extracellular matrix further suggested roles in extracellular remodeling and intercellular communication (**Figure 2D**). The GO Molecular Function (MF) analysis revealed significant enrichment in cysteine-type peptidase activity and its regulatory functions, binding activities related to cadherin, laminin, collagen, and extracellular matrix components, as well as antigen binding and NAD binding, indicating potential involvement in proteolytic processes, cell adhesion interactions, immune recognition, and metabolic regulation (**Figure 2E**). KEGG pathway analysis showed that the DEPs were significantly associated with arginine biosynthesis, glycolysis/gluconeogenesis, carbon metabolism, biosynthesis of amino acids, and apoptosis, highlighting potential roles in metabolic reprogramming and cell death regulation (**Figure 2F**).

To systematically investigate the biological functions of these 70 DEPs, PPI network was constructed using the STRING database and visualized using Cytoscape. Based on the Cytoscape analysis, ten key hub proteins with high network scores were identified: CTSS, CTSL, ANPEP, MPO, GAPDH, ADIPOQ, CTTN, HMGB1, G6PD, and BGN (**Figure 2G**). Furthermore, GSEA pathway enrichment analysis revealed significant enrichment of proteins in signaling pathways such as interleukin signaling, cellular response to stimuli, and the complement system (**Figure 2H**).

### 3.3 Serum metabolomic features of HCC and non-HCC populations

**Figure 3:**
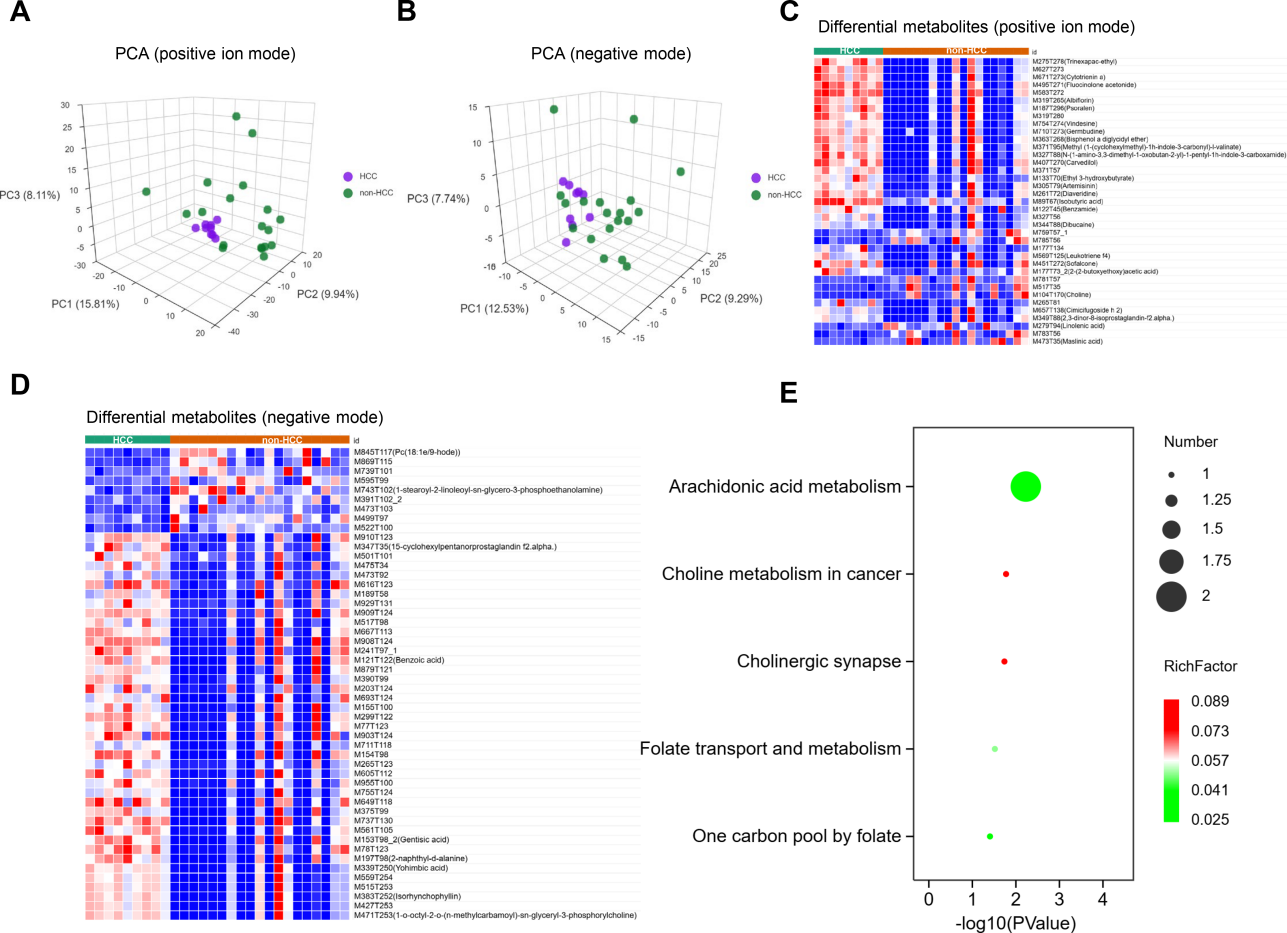
Overall characteristics of serum metabolites. **(A)** PCA score plot (positive-ion mode). **(B)** PCA score plot (negative mode). **(C)** Heatmap of serum metabolomic features in HCC vs non-HCC (positive-ion mode). **(D)** Heatmap of serum metabolomic features in HCC vs non-HCC (negative mode). **(E)** KEGG pathway analyses with DEMs.

PCA of the metabolites detected in both positive and negative ionization modes demonstrated significant differences in metabolic profiles between the HCC and non-HCC groups, thereby enabling effective group separation (**Figures 3A, B**). The heatmap displays the relative abundances of differential metabolites detected (VIP > 1, adjusted *P* value < 0.05 and |log_1.5_(FC)| >1) in HCC and non-HCC cohorts (**Figures 3C, D**). Using KEGG enrichment analysis, we further explored the potential biological functions of these DEMs. The significantly enriched pathways were mainly associated with arachidonic acid metabolism, choline metabolism in cancer, cholinergic synapse signaling, folate transport and metabolism, and the one-carbon pool by folate pathway, suggesting potential involvement in lipid metabolism, cancer-related metabolic reprogramming, and one-carbon metabolic processes (**Figure 3E**).

### 3.4 Correlation and hierarchical clustering of DEPs and DEMs

**Figure 4:**
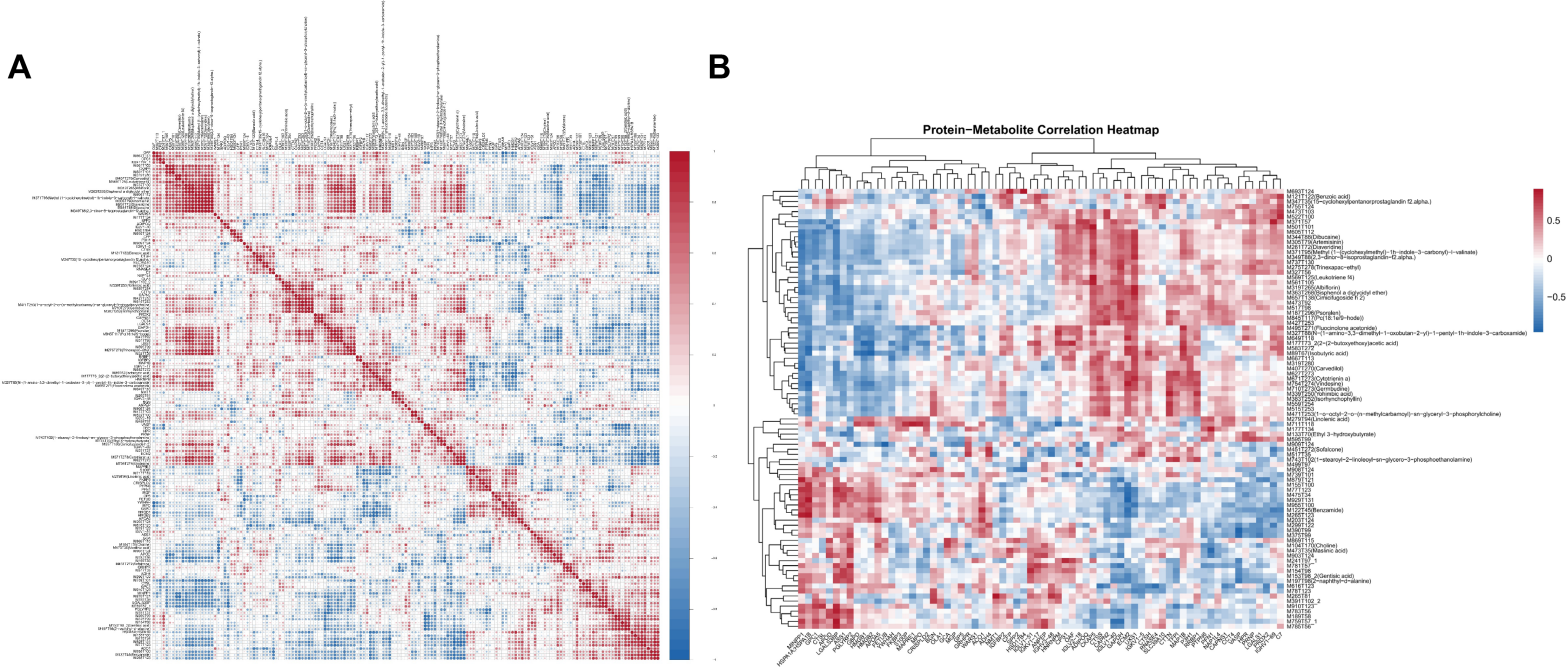
Correlation and hierarchical clustering analysis of DEPs and DEMs. **(A)** Correlation coefficient matrix heatmap showing the relationships between DEPs and DEMs. Red indicates positive correlations, whereas blue indicates negative correlations. **(B)** Hierarchical clustering heatmap based on correlation analysis between DEPs and DEMs, illustrating clustering patterns according to similarity in correlation profiles.

After the preliminary screening of DEPs and DEMs, correlation analysis was performed to explore their potential associations. The correlation coefficient matrix heatmap demonstrated distinct positive and negative correlations between DEPs and DEMs, suggesting coordinated alterations at the proteomic and metabolomic levels (**Figure 4A**). Furthermore, hierarchical clustering analysis based on correlation patterns revealed that these molecules could be grouped into several clusters with similar correlation profiles, indicating potential functional interactions and shared regulatory mechanisms (**Figure 4B**).

However, further pathway enrichment analysis showed that differential proteins and differential metabolites did not share any significantly co-enriched pathways, suggesting that although correlation patterns were observed, they may not converge on common biological pathways.

### 3.5 Screening of serum protein biomarkers for HCC

**Figure 5:**
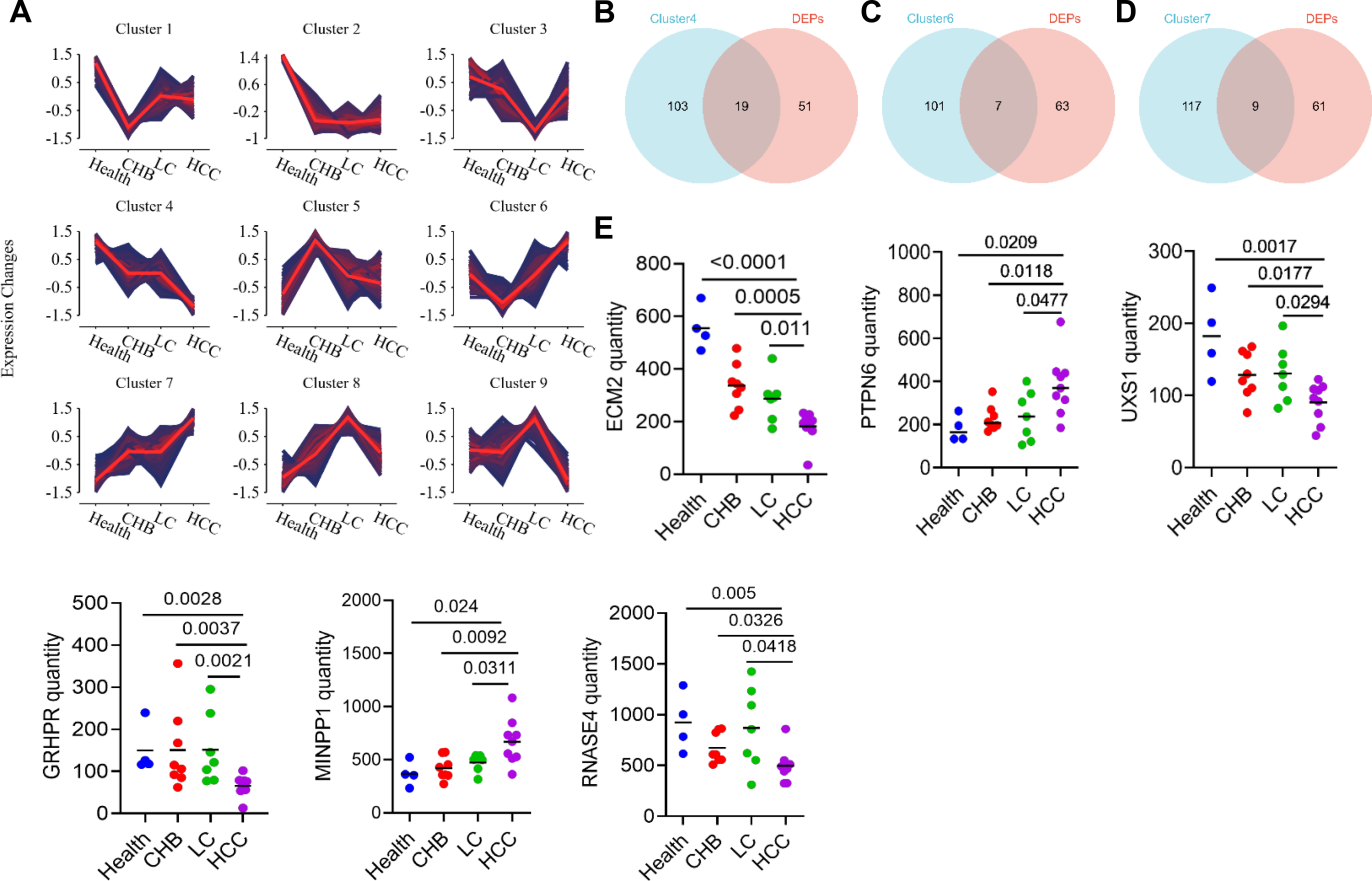
Screening of potential serum protein markers for HCC. **(A)** Trend clustering plot. **(B)** Venn diagram of Cluster4 and DEPs. **(C)** Venn diagram of Cluster6 and DEPs. **(D)** Venn diagram of Cluster7 and DEPs. **(E)** The expression of ECM2, PTPN6, UXS1, GRHPR, MINPP1 and RNASE4 in the four groups.

To investigate dynamic changes in protein expression across multiple sample groups, we performed a fuzzy clustering analysis on DEPs to identify those exhibiting progressive increases or decreases in expression across the Health, CHB, LC, and HCC groups. The results revealed that clusters 6 and 7 contained proteins that were highly expressed in HCC, while cluster 4 contained proteins that were downregulated in HCC (**Figure 5A**). Venn diagram analyses were then conducted between the selected DEPs and the HCC-upregulated or HCC-downregulated proteins in clusters 4, 6 and 7. This led to the identification of 35 DEPs that were altered in a similar way in all three clusters (**Figures 5B–D**). Proteins showing differential expression across groups (*P* < 0.05) included ECM2, PTPN6, UXS1, GRHPR, MINPP1, RNASE4 (**Figure 5E**).

### 3.6 Screening of serum metabolic biomarkers for HCC

**Figure 6:**
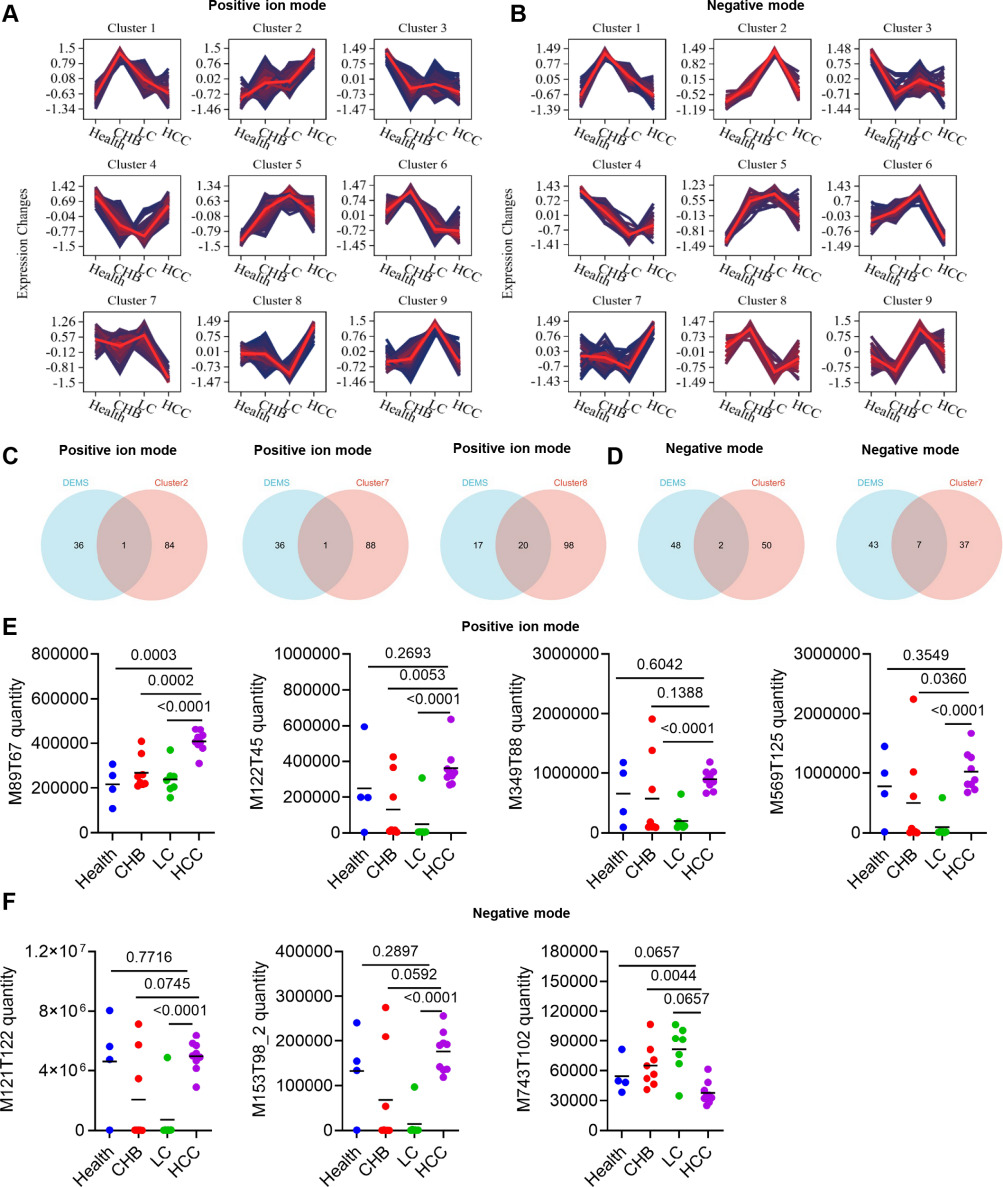
Screening of potential serum metabolite biomarkers for HCC. **(A)** Trend Clustering Plot Across Health, CHB, LC, and HCC Groups in positive-ion mode. **(B)** Trend Clustering Plot Across Health, CHB, LC, and HCC Groups in negative mode. **(C, D)** Venn diagram of Clusters and DEMs. **(E)** Expression levels of significantly altered metabolites in positive-ion mode across the four groups. **(F)** Expression levels of significantly altered metabolites in negative mode across the four groups.

Fuzzy c-means clustering was conducted on the differential metabolites identified in both positive and negative-ion modes across the Health, CHB, LC, and HCC groups (**Figures 6A, B**). Subsequently, differential metabolites exhibiting increasing or decreasing trends specifically in the HCC group (positive-ion mode: clusters 2, 7, and 8; negative mode: clusters 6 and 7) were selected and subjected to an intersection analysis with those showing differences between the HCC and non-HCC groups. This process identified 22 differential metabolites in the positive-ion mode and 9 in the negative mode (**Figures 6C, D**). Finally, by restricting the analysis to endogenous metabolites and selecting those that showed significant differences (*P* < 0.01) between the HCC group and each of the Health, CHB, and LC groups, we ultimately identified the metabolite M89T67 (isobutyric acid) (**Figures 6E, F**).

### 3.7 Validation of differential expression and prognostic significance of GRHPR in HCC

**Figure 7:**
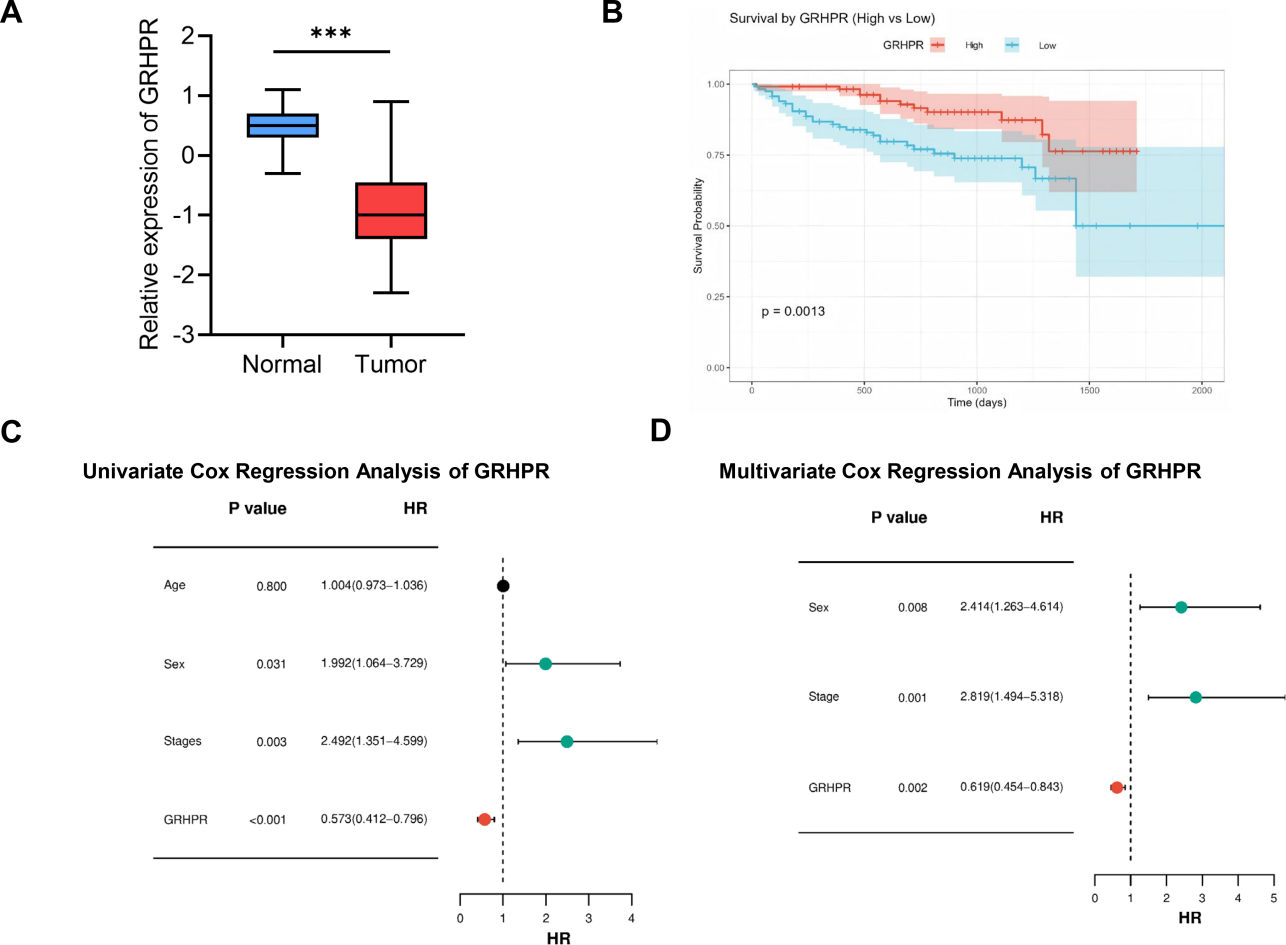
GRHPR Downregulation and Independent Prognostic Value in HCC. **(A)** Validation of GRHPR protein expression using CPTAC proteomics data from the HPA. **(B)** Kaplan-Meier survival analysis of GRHPR. **(C)** Univariate Cox regression analysis of prognostic factors in HCC patients. **(D)** Multivariate Cox regression analysis of prognostic factors in HCC patients.

To validate the differential expression of candidate proteins, we analyzed proteomic data from 330 HCC samples obtained from the HPA, based on the CPTAC cohort. Compared with normal tissues, GRHPR expression was significantly reduced in HCC tissues (**Figure 7A**). To further evaluate the prognostic value of GRHPR, clinical information and GRHPR expression data from 231 patients in the HPA validation cohort were analyzed. Kaplan–Meier survival analysis demonstrated that patients with high GRHPR expression had significantly improved overall survival compared to those with low expression (*P* < 0.05), suggesting that GRHPR may serve as a favorable prognostic biomarker in HCC (**Figure 7B**). Univariate Cox regression analysis identified gender, tumor stage, and GRHPR expression as significant prognostic factors (**Figure 7C**). Multivariate Cox regression analysis further confirmed that gender, tumor stage, and GRHPR expression remained independently associated with overall survival, indicating that GRHPR is an independent prognostic indicator in HCC (**Figure 7D**).

### 3.8 Diagnostic performance of protein and metabolite biomarkers in HCC

**Figure 8:**
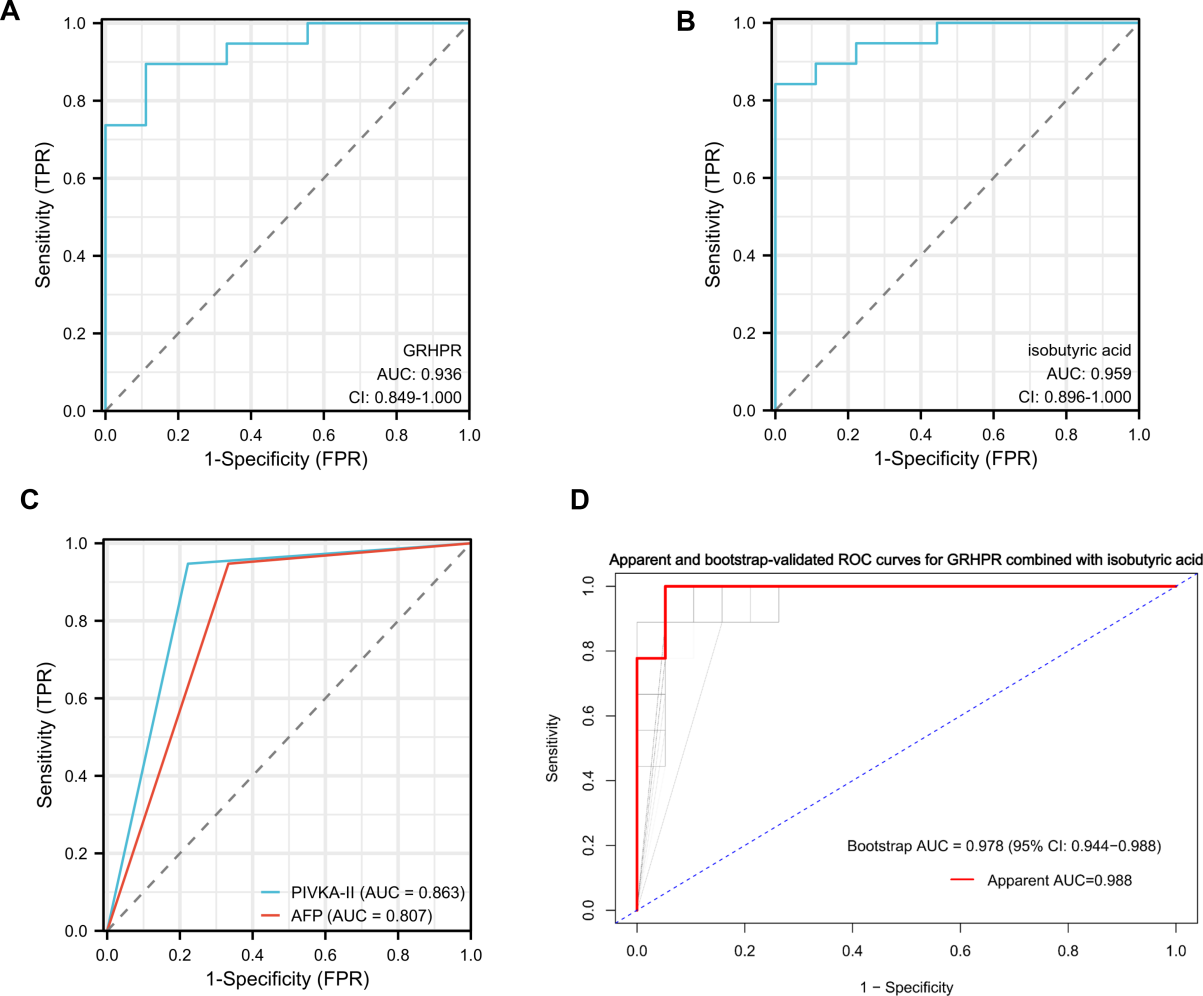
Clinical performance of protein biomarkers in HCC diagnosis. **(A)** ROC analysis of GRHPR. **(B)** ROC analysis of isobutyric acid. **(C)** ROC analysis of AFP, PIVKA-II. **(D)** bootstrap-corrected ROC curves for GRHPR and isobutyric acid combined.

To further evaluate the diagnostic potential of GRHPR and isobutyric acid, we performed ROC curve analyses. GRHPR showed an AUC of 0.936 and demonstrated 89.47% sensitivity and 88.89% specificity (**Figure 8A**). The differential metabolite isobutyric acid also showed good diagnostic performance, with an AUC of 0.959 (**Figure 8B**). ROC analyses were performed for PIVKA-II and AFP using cutoff values of PIVKA-II > 40 mAU/mL and AFP > 20 ng/mL to evaluate their diagnostic performance. PIVKA-II achieved an AUC of 0.863, and AFP reached an AUC of 0.807 (**Figure 8C**). The joint diagnostic performance of GRHPR and isobutyric acid was further evaluated. The combined model achieved an AUC of 0.988, with a bootstrap-corrected AUC of 0.978, suggesting a modest improvement in distinguishing HCC compared with a single marker alone (**Figure 8D**).

## 4 Discussion

In this study, we systematically profiled circulating proteins and metabolites across the liver disease spectrum and identified biologically coherent and diagnostically relevant signatures for HCC. Network analysis of 70 DEPs identified ten high-scoring hub proteins within the protein-protein interaction network, and GSEA revealed significant enrichment in interleukin signaling, cellular response to stimuli, and complement cascade pathways implicated in tumor-immune crosstalk. Proteomic screening further identified six candidate proteins.

Taken together, the observed pathway and network signals provide a plausible explanatory framework. The enrichment of interleukin and complement signaling pathways aligns with the presence of an inflamed, tumor-promoting microenvironment, where immune signaling, opsonization, and cytolytic processes contribute to tumor progression and immune evasion (12–15). The hub-based topology of the PPI subnetwork suggests the presence of a coordinated regulatory mechanism, in contrast to a series of independent molecular changes (16–18). Functionally, ECM2 is a matrix glycoprotein involved in extracellular matrix remodeling and tissue structural organization (19). PTPN6 is implicated in immune receptor signaling (20). UXS1, MINPP1, and GRHPR are linked to carbohydrate metabolism, inositol phosphate signaling, and glyoxylate metabolic pathways (21–23). In conjunction with the observed metabolic shifts, these molecular alterations indicate a coordinated reprogramming of immune and metabolic processes in HCC, thereby providing a biological basis for their diagnostic and prognostic utility.

In this study, we observed that GRHPR protein levels were significantly reduced in patients with HCC, which is consistent with previous findings at the tissue level and further supports its potential involvement in metabolic reprogramming during hepatocarcinogenesis (24). GRHPR is a key oxidoreductase involved in glyoxylate and hydroxypyruvate metabolism and plays an essential role in maintaining intracellular glyoxylate homeostasis and redox balance (25). Reduced expression of GRHPR may lead to abnormal accumulation of metabolic substrates or disruption of related metabolic pathways, thereby affecting tumor cell energy metabolism and biosynthetic processes. Previous studies have suggested that tumor cells reprogram amino acid and organic acid metabolism to sustain rapid proliferation, and the downregulation of GRHPR may represent a component of this metabolic adaptation.

Unlike prior studies that primarily focused on tumor tissues, our study innovatively applied proteomic techniques to detect GRHPR protein levels in serum samples. Serum collection is relatively simple and minimally invasive, offering high clinical accessibility and making it more suitable for large-scale screening and longitudinal monitoring. Compared with invasive tissue biopsy procedures, serum-based detection is more feasible for clinical implementation, particularly in patient populations where repeated tissue sampling is impractical.

Researchers have found that isobutyric acid promotes colorectal cancer metastasis by activating RACK1. In addition, fecal isobutyric acid levels in patients with HCC are significantly higher than those in healthy individuals (26, 27). In this study, we found that isobutyric acid levels were increased in the serum of patients with HCC, suggesting that it may serve as a potential circulating biomarker for HCC. Moreover, a combined diagnostic model integrating isobutyric acid and GRHPR achieved a bootstrap-corrected AUC of 0.978, indicating favorable discriminatory performance. This panel may therefore have potential utility as an adjunct to ultrasound-based screening and as a triage tool to help prioritize patients for subsequent confirmatory imaging.

This study employed multi-omics profiling across multiple clinical states (Health, CHB, LC, and HCC), enabling the rigorous identification of metabolites that distinguish HCC from each comparator group. The integration of proteomic markers with survival outcomes further supports their potential utility in risk stratification beyond diagnostic applications.

Nevertheless, several limitations should be acknowledged. Previous studies have demonstrated that hepatocellular injury and chronic inflammation in cirrhosis are associated with substantial alterations in circulating proteomic and metabolomic profiles (28, 29). In our study, analysis comparing high and low ALT groups showed no significant differences in GRHPR or isobutyric acid levels, suggesting that liver function status had a limited impact on their performance as serum biomarkers for HCC (**Supplementary Figure S1**). However, a residual confounding effect cannot be entirely excluded. Larger, multicenter studies are needed to validate these findings and to further clarify tumor-specific alterations in diverse patient populations.

## 5 Conclusion

Using serum proteomic and metabolomic detection, this study identified circulating molecular changes associated with hepatocarcinogenesis. Systems analyses (PPI and GSEA) highlighted dysregulated inflammatory signaling, complement activity, and extracellular-matrix remodeling, providing biological context for the observed markers. he protein GRHPR and the metabolite isobutyric acid effectively distinguished HCC from non-HCC groups, and GRHPR additionally demonstrated prognostic relevance. These results suggest that serum proteomic and metabolomic testing could enhance early detection and risk stratification in HCC. As multicenter validation was not performed, future work should confirm these candidates with orthogonal assays in larger, etiology-diverse cohorts and evaluate clinical utility in prospective settings.

## Data Availability

The mass spectrometry proteomics data have been deposited to the ProteomeXchange Consortium via the iProX partner repository (https://proteomecentral.proteomexchange.org) with the dataset identifier PXD076855. The metabolomics data have been deposited in the MetaboLights repository (https://www.ebi.ac.uk/metabolights/MTBLS14248) with the study identifier MTBLS14248.
