# Supplementary material for: Serum proteomics and metabolomics reveal novel noninvasive molecular signatures for hepatocellular carcinoma diagnosis
Source: Front Oncol. 2026 Apr 22;16:1797408. doi: 10.3389/fonc.2026.1797408 (PMC13143717; doi:10.3389/fonc.2026.1797408)
Supplement: Supplementary file 1 [file DataSheet1.docx]

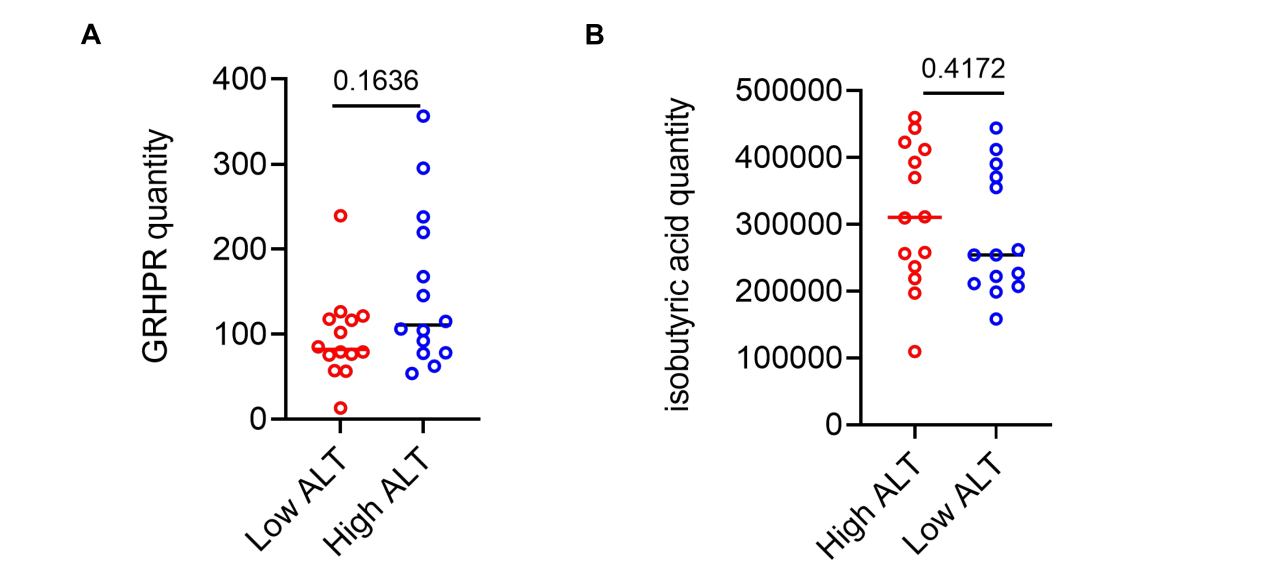


**Figure S1**

Comparison of GRHPR and isobutyric acid levels between high and low ALT groups. (A) Comparison of GRHPR levels between high ALT and low ALT groups. (B) Comparison of isobutyric acid levels between high ALT and low ALT groups.
