# Supplementary material for: Serum proteomics and metabolomics reveal novel noninvasive molecular signatures for hepatocellular carcinoma diagnosis
Source: Front Oncol. 2026 Apr 22;16:1797408. doi: 10.3389/fonc.2026.1797408 (PMC13143717; doi:10.3389/fonc.2026.1797408)
Supplement: Supplementary file 2 [file Table1.docx]

Clinical information of the Health, CHB, and LC groups

**Table S1. characteristics of the non-HCC population**

| Sample | Age | Gender | HBsAg | HBeAg | ALT(U/L) | AST(U/L) | PIVKA-II  (mAU/mL) | AFP（ng/ml） |
| --- | --- | --- | --- | --- | --- | --- | --- | --- |
| NC1 | 19 | female | Negative | Positive | 6 | 15 | 22 | 2.49 |
| NC2 | 33 | female | Negative | Positive | 10 | 15 | 19 | 2.57 |
| NC3 | 37 | male | Negative | Positive | 41 | 25 | 21 | 3.3 |
| NC4 | 51 | male | Negative | Positive | 10 | 18 | 31 | 2.12 |
| CHB1 | 30 | female | Positive | Negative | 48 | 36 | 32 | 2.16 |
| CHB2 | 17 | male | Positive | Negative | 56 | 59 | 25 | 1.06 |
| CHB3 | 33 | male | Positive | Negative | 305 | 97 | 25 | 5.56 |
| CHB4 | 33 | female | Positive | Negative | 168 | 75 | 25 | 2.83 |
| CHB5 | 53 | female | Positive | Negative | 63 | 25 | 24 | 4.53 |
| CHB6 | 67 | female | Positive | Positive | 773 | 1001 | 25 | 1.66 |
| CHB7 | 33 | male | Positive | Positive | 823 | 288 | 72 | 7.39 |
| CHB8 | 35 | male | Positive | Positive | 61 | 36 | 33 | 6.66 |
| LC1 | 27 | male | Positive | Negative | 54 | 38 | 27 | 3.19 |
| LC2 | 65 | female | Positive | Negative | 37 | 41 | 12 | 6.4 |
| LC3 | 47 | male | Positive | Negative | 61 | 57 | 23 | 5.6 |
| LC4 | 61 | male | Positive | Negative | 92 | 144 | 18 | 69.8 |
| LC5 | 49 | female | Positive | Positive | 67 | 96 | 20 | 7.06 |
| LC6 | 66 | female | Positive | Positive | 35 | 34 | 22 | 2.76 |
| LC7 | 59 | female | Positive | Positive | 1093 | 1019 | 35 | 14 |

Clinical information of the HCC group.

**Table S2. characteristics of the HCC population**

| Sample | Age | Gender | HBsAg | HBeAg | ALT(U/L) | AST(U/L) | PIVKA-II  (mAU/mL) | AFP（ng/ml） |
| --- | --- | --- | --- | --- | --- | --- | --- | --- |
| HCC1 | 37 | male | Positive | Positive | 29 | 23 | 353 | 13957 |
| HCC2 | 51 | male | Positive | Negative | 33 | 32 | 20 | 4.47 |
| HCC3 | 53 | male | Positive | Negative | 123 | 86 | 58.8 | 8.81 |
| HCC4 | 53 | male | Positive | Negative | 38 | 34 | 2527 | 7.43 |
| HCC5 | 18 | male | Positive | Negative | 33 | 26 | 64 | 30128 |
| HCC6 | 44 | female | Positive | Negative | 85 | 67 | 237 | 29.7 |
| HCC7 | 48 | male | Positive | Negative | 51 | 43 | 75000 | 34440 |
| HCC8 | 51 | male | Positive | Negative | 42 | 38 | 23 | 89.1 |
| HCC9 | 47 | female | Positive | Negative | 27 | 28 | 94 | 22 |
